# Exocytosis in mouse vestibular Type II hair cells shows a high‐order Ca^2+^ dependence that is independent of synaptotagmin‐4

**DOI:** 10.14814/phy2.14509

**Published:** 2020-07-20

**Authors:** Paolo Spaiardi, Walter Marcotti, Sergio Masetto, Stuart L. Johnson

**Affiliations:** ^1^ Department of Brain and Behavioral Sciences University of Pavia Pavia Italy; ^2^ Department of Biomedical Science University of Sheffield Sheffield UK

**Keywords:** Exocytosis, Ribbon Synapse, Synaptotagmin‐4, Vestibular Hair Cell

## Abstract

Mature hair cells transduce information over a wide range of stimulus intensities and frequencies for prolonged periods of time. The efficiency of such a demanding task is reflected in the characteristics of exocytosis at their specialized presynaptic ribbons. Ribbons are electron‐dense structures able to tether a large number of releasable vesicles allowing them to maintain high rates of vesicle release. Calcium entry through rapidly activating, non‐inactivating Ca_V_1.3 (L‐type) Ca^2+^ channels in response to cell depolarization causes a local increase in Ca^2+^ at the ribbon synapses, which is detected by the exocytotic Ca^2+^ sensors. The Ca^2+^ dependence of vesicle exocytosis at mammalian vestibular hair cell (VHC) ribbon synapses is believed to be linear, similar to that observed in mature cochlear inner hair cells (IHCs). The linear relation has been shown to correlate with the presence of the Ca^2+^ sensor synaptotagmin‐4 (Syt‐4). Therefore, we studied the exocytotic Ca^2+^ dependence, and the release kinetics of different vesicle pool populations, in Type II VHCs of control and *Syt‐4* knockout mice using patch‐clamp capacitance measurements, under physiological recording conditions. We found that exocytosis in mature control and knockout Type II VHCs displayed a high‐order dependence on Ca^2+^ entry, rather than the linear relation previously observed. Consistent with this finding, the Ca^2+^ dependence and release kinetics of the ready releasable pool (RRP) of vesicles were not affected by an absence of Syt‐4. However, we did find that Syt‐4 could play a role in regulating the release of the secondary releasable pool (SRP) in these cells. Our findings show that the coupling between Ca^2+^ influx and neurotransmitter release at mature Type II VHC ribbon synapses is faithfully described by a nonlinear relation that is likely to be more appropriate for the accurate encoding of low‐frequency vestibular information, consistent with that observed at low‐frequency mammalian auditory receptors.

## Graphical abstract

Here we show that the coupling between neurotransmitter release at the ribbon synapses of mature vestibular Type II hair cells display a high‐order dependence on calcium influx. The calcium dependence was not affected by an absence of the calcium‐sensing synaptic molecule synaptotagmin‐4, which has been shown to be involved in establishing the linear calcium dependence of high‐frequency auditory hair cells. Our findings suggest that a nonlinear exocytotic calcium dependence in vestibular hair cells is likely to be more appropriate for the accurate encoding of low‐frequency vestibular information.

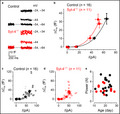

## 1 INTRODUCTION

The transfer of information at all chemical synapses relies on a highly coordinated mechanism of Ca^2+^‐dependent fusion of docked vesicles at the presynaptic membrane. At conventional synapses, where neurotransmission is locked to presynaptic action potentials (APs), signal transfer is discrete and can be efficiently sustained by a relatively small readily releasable pool (RRP) of synaptic vesicles (Matthews & Fuchs, 2010). By contrast, at most sensory cell synapses, neurotransmitter release is graded to accurately represent stimulus intensity, is maintained throughout the duration of the stimulus, and occurs spontaneously at rest in the absence of any external stimulus. This applies to the sensory hair cells of the mammalian auditory and vestibular systems, which encode sound or head movements, respectively, with great precision. Vestibulo‐ocular reflexes are likely the fastest reflexes in the body that drive eye muscles to move the eye opposite to head motion in order to maintain gaze. In primates, eye motion nearly perfectly compensates for head motion at frequencies from below 1 Hz up to at least 25 Hz (Huterer & Cullen, 2002). To achieve this demanding task, vesicle release in vestibular hair cells (VHCs) occurs at specialized ribbon synapses similar to those in the auditory and visual systems (Matthews & Fuchs, 2010). Synaptic ribbons are presynaptic electron‐dense organelles able to tether large distinct pools of synaptic vesicles, which allow the hair cells to provide rapid and relatively inexhaustible release of neurotransmitter in response to fast and prolonged stimulation (Lysakowski & Goldberg, 1997; Matthews & Fuchs, 2010; Pangrsic, Singer, & Koschak, 2018; Parsons, Lenzi, Almers, & Roberts, 1994; Roberts, Jacobs, & Hudspeth, 1990; Smith & Sjostrand, 1961).

Immature cochlear inner hair cells (IHCs) fire spontaneous APs that are thought to drive activity in the auditory pathway before the onset of sound‐evoked activity (Clause et al., 2014; Kros, Ruppersberg, & Rüsch, 1998). To faithfully preserve the timing and pattern of AP activity and ensure that vesicles are predominantly released at the peak of the AP rather than at interspike intervals, the functional coupling between Ca^2+^ influx and exocytosis at these immature IHC ribbon synapses is high‐order (Johnson, Franz, Knipper, & Marcotti, 2009; Johnson et al., 2010; Johnson, Marcotti, & Kros, 2005), similar to conventional synapses (Augustine, Charlton, & Smith, 1985; Dodge & Rahmimoff, 1967). With the onset of hearing at postnatal day 12 (P12), IHCs lose the ability to fire APs and instead respond to sound with graded and sustained receptor potentials that encode sound intensity and stimulus envelope with the precision required for accurate sound localization (Kros et al., 1998). To achieve such remarkable precision, the Ca^2+^ dependence of exocytosis in mature high‐frequency IHCs becomes linear (Brandt, Khimich, & Moser, 2005; Johnson, Forge, Knipper, Münkner, & Marcotti, 2008; Johnson et al., 2005, 2009, 2010), which is likely to enable IHCs to extend their overall dynamic range allowing the representation of different sound intensities (Johnson et al., 2005). The Ca^2+^ binding protein, otoferlin, has been implicated as the Ca^2+^ sensor for graded neurotransmitter exocytosis at ribbon synapses of both vestibular (Dulon, Safieddine, Jones, & Petit, 2009) and cochlear hair cells (Roux et al., 2006; Vincent, Bouleau, Safieddine, Petit, & Dulon, 2014). However, it is the expression of another Ca^2+^ sensor, synaptotagmin‐4 (Syt‐4), that has been shown to be essential for establishing the linear Ca^2+^ dependence of exocytosis in mature IHCs at the onset of hearing (Johnson et al., 2010). Syt‐4 seems not to be functionally involved in cochlear hair cells where exocytosis shows a high‐order Ca^2+^ dependence, such as in immature IHCs and verylow‐frequency adult gerbil IHCs (Johnson et al., 2008, 2010).

Vestibular sensory epithelia, such as the utricle and saccule, contain two types of sensory hair cell known as Type I and Type II cells. Unlike the clear functional division of cochlear IHCs and outer hair cells (OHCs), that of the different VHC types is still uncertain. Both VHC types release vesicles at ribbon synapses onto afferent terminals (Bonsaquet, Brugeaud, Compan, Desmadryl, & Chabbert, 2006; Dulon et al., 2009; Kirk, Meredith, Benke, & Rennie, 2017; Sadeghi, Pyott, Yu, & Glowatzki, 2014; Songer & Eatock, 2013). Type I VHCs are enclosed by a single giant calyx synaptic terminal confining an intercellular compartment inside which K^+^ can accumulate (Contini et al., 2012; Lim, Kindig, Donne, Callister, & Brichta, 2011; Spaiardi et al., 2019) and modulate afferent transmission (Contini, Price, & Art, 2017). By contrast, Type II cells are contacted by several bouton‐like terminals (Lysakowski & Goldberg, 1997), making them more similar in terms of synaptic architecture to cochlear IHCs. Another similarity to mature IHCs is that VHCs respond to head motion with graded receptor potentials and are also thought to have a linear relationship between Ca^2+^ influx and neurotransmitter release that seemed to be related to the presence of the hair cell synaptic Ca^2+^ sensor otoferlin (Dulon et al., 2009; Vincent et al., 2014). However, in VHCs, the spontaneous release of neurotransmitter was seemingly independent of the presence of otoferlin (Dulon et al., 2009). To investigate whether Syt‐4 was also involved in determining the linear relation between Ca^2+^ entry and neurotransmitter release in mature VHCs, we compared the Ca^2+^ dependence and kinetics of vesicle release in Type II VHCs from control (wild‐type and *Syt‐4^+/−^*) and *Syt‐4* knockout (*Syt‐4^−/−^*) mice (Ferguson, Anagnostaras, Silva, & Herschman, 2000). We focused on the Type II cells since these rely purely on chemical synaptic transmission and have similar synaptic architecture to the boutons of cochlear hair cells. Whole‐cell patch‐clamp recordings were performed on cells maintained in approximately physiological conditions, that is 1.3‐mM extracellular Ca^2+^ and body temperature. In contrast to a previous report on more immature VHCs (Dulon et al., 2009), we found that mature Type II VHCs exhibited a high‐order relation between Ca^2+^ influx and neurotransmitter exocytosis. As expected from a nonlinear Ca^2+^ dependence of exocytosis, we found that Syt‐4 was not involved in determining this relation nor was it involved in the exocytosis of the RRP of synaptic vesicles at Type II VHC ribbon synapses. However, we did find that Syt‐4 could play a role in regulating the release of the secondary releasable pool (SRP) in these cells. These findings are consistent with Syt‐4 being essential only for the linearly Ca^2+^‐dependent exocytosis in mature high‐frequency hair cells (Johnson et al., 2010).

## 2 MATERIALS AND METHODS

Vestibular Type II hair cells (*n* = 33) from control (*Syt‐4^+/+^* and *Syt‐4^+/−^*) and *Syt‐4* knockout (*Syt‐4^−/−^*; Ferguson et al., 2000) mice were studied in acutely dissected utricles from postnatal day 11 (P11) to P28, where the day of birth is P0.

Animals of either sex were killed by cervical dislocation, under schedule 1 in accordance with the UK Home Office regulations under the Animals (Scientific Procedures) Act 1986 and following approval by the University of Sheffield Ethical Review Committee. Mouse utricles were dissected in normal extracellular solution (in mM): 135 NaCl, 5.8 KCl, 1.3 CaCl_2_, 0.9 MgCl_2_, 0.7 NaH_2_PO_4_, 5.6 D‐glucose, and 10 Hepes‐NaOH. Sodium pyruvate (2 mM), MEM amino acids solution (50X, without L‐Glutamine), and MEM vitamins solution (100X) were added from concentrates (Fisher Scientific, UK). The pH was adjusted to 7.5 (osmolality ~ 308 mmol/kg). The dissected utricles were transferred to a microscope chamber, immobilized using a nylon mesh fixed to a stainless steel ring, and continuously perfused with the above extracellular solution. The utricles were observed with an upright microscope (Nikon FN1, Japan) equipped with Nomarski differential interference contrast optics (X60 water immersion objective and X15 eyepieces).

### 2.1 Identification of hair cell type

Vestibular sensory epithelia contain two types of sensory receptors, called Type I and Type II hair cells, which differ in shape, innervation pattern, and electrophysiological properties (Eatock & Songer, 2011). The acutely dissected preparation used for our recordings prevented the identification of VHCs based on their morphological properties. A characteristic electrophysiological feature of Type I hair cells is the expression of a low‐voltage activated outwardly rectifying K^+^ current (*I*_K,L_) (Rennie & Correia, 1994; Rüsch & Eatock, 1996). Although the inward rectifying mixed Na^+^/K^+^ current *I*_h_ has been suggested to be a specific marker for Type II hair cells (Eatock & Rüsch, 1997), a recent study has demonstrated that most utricle Type I hair cells also express *I*_h_ (Horwitz, Risner‐Janiczek, Jones, & Holt, 2011). Therefore, in a first set of experiments we recorded from VHCs using a Cs‐Glutamate‐based intracellular solution that allowed us to see the *I*_K,L_ and thus identify Type I hair cells (Bao, Wong, Goldberg, & Eatock, 2003). In contrast to most voltage‐gated K^+^ conductances, *G*_K,L_ is significantly permeable to Cs^+^ (Rennie & Correia, 2000). For this study, we focused on the Type II cells since they are known to rely entirely on traditional chemical synaptic transmission to transfer information to afferent fibers and have similar synaptic architecture to the boutons of cochlear hair cells. We have an additional study that is based on the mixed chemical and non‐quantal transmission in Type I VHCs. Immediately following Type II hair cell identification by the absence of *I*_K,L_, to isolate the Ca^2+^ current (*I*_Ca_), we perfused the cell with an extracellular solution containing 4‐AP, TEA, and Cs^+^ (see composition below) which blocks the majority of *I*_K,L_, other K^+^ currents, and *I*_h_, respectively (Rennie & Correia, 1994). From these experiments we found that only mature Type II VHCs showed a robust change in membrane capacitance (Δ*C*_m_) in response to an *I*_Ca_, whereas Type I cells showed a large transient Δ*C*_m_ that increased with depolarization (unpublished observations). In later experiments, we modified the intracellular solution (see composition below) by adding 4‐AP and TEA to block *I*_K,L_ and other K^+^ currents (Rennie & Correia, 1994; Rennie, Ricci, & Correia, 1996) from the beginning of the recording and exploited the absence of the transient Δ*C*_m_ to identify Type II hair cells.

### 2.2 Electrophysiology

Whole‐cell patch‐clamp recordings were performed at body temperature (34–37 ºC) using an Optopatch (Cairn Research Ltd, UK) amplifier. Patch pipettes (3–4 MΩ) were coated with surf wax (Mr. Zogs SexWax, USA) to minimize the fast capacitance transient of the patch pipette.

For experiments where *I*_K,L_ was not immediately blocked, the pipette intracellular solution contained (in mM): 106 Cs‐glutamate, 20 CsCl, 3 MgCl2, 1 EGTA‐CsOH, 5 Na_2_ATP, 0.3 Na_2_GTP, 5 HEPES‐CsOH, and 10 Na_2_‐phosphocreatine (pH 7.3; 294 mmol/kg). In these experiments, after establishing the VHC type, the remaining K^+^ currents and *I*_h_ were blocked by locally perfusing extracellular solution containing (in mM): 110 NaCl, 5.8 CsCl, 1.3 CaCl_2_, 0.9 MgCl_2_, 0.7 NaH_2_PO_4_, 5.6 D‐glucose, 10 Hepes, 30‐mM TEA, and 15‐mM 4‐AP. The pH was adjusted to 7.5 (osmolality ~ 312 mmol/kg).

For experiments where *I*_K,L_ and other K^+^ currents were blocked from the beginning of the recording by blockers in the intracellular solution, the pipette intracellular solution contained (in mM): 125 CsCl, 3 MgCl_2_, 1 EGTA‐CsOH, 5 Na_2_ATP, 5 Hepes‐CsOH, 5 TEA, and 5 4‐AP (pH 7.3; 294 mmol/kg). In this case, the acutely isolated utricles were continuously bath‐perfused with the standard extracellular solution. Data for *I*_Ca_ and cell membrane capacitance obtained by the two above sets of experiments were pooled together.

Data acquisition was controlled by pClamp software using a Digidata 1440A board (Molecular Devices, USA). The capacitance signal was amplified (50x), filtered at 250 Hz, and sampled at 5 kHz. Voltage‐clamp recordings were low‐pass filtered at 2.5 kHz (8‐pole Bessel) and sampled at 5 kHz. Data analysis was performed using Clampfit (Molecular Devices, USA) and Origin software (OriginLab, USA).

To study the voltage dependence of *I*_Ca_, the peak current was measured at different membrane potentials and the resulting *I‐V* relation was fitted with the following equation:

(1) $$I\left(v\right)=\frac{g_{\max}\left(V‐V_{\text{rev}}\right)}{\text{1}\;+\;\exp\left(\frac{V_{1/2}‐V}{S}\right)}$$

where *V* is the membrane potential, *V*_rev_ is the reversal potential, *g*_max_ is the maximum chord conductance, *V*_½_ is the membrane potential at which the conductance is half activated, and *S* is the voltage change per *e*‐fold increase of *I(V)*.

Real‐time measurement of cell membrane capacitance was performed with the “track‐in” circuitry of the Optopatch amplifier (Johnson, Thomas, & Kros, 2002) using a 4 kHz sine wave voltage command (13 mV RMS) applied at the holding potential of −84 mV or −81 mV. The exocytosis of synaptic vesicles was measured as the change in membrane capacitance (Δ*C*_m_) produced by Ca^2+^ influx elicited by depolarizing voltage steps of variable intensity and duration. The sine wave used to measure real‐time *C*_m_ was interrupted for the duration of the depolarizing voltage steps. The Δ*C*_m_ as a function of cell membrane voltage was obtained as the difference between the mean baseline capacitance signal and that measured over a 200 ms, or greater, period after each depolarizing voltage step. The Ca^2+^ dependence of vesicle exocytosis was assessed by fitting the variation in Δ*C*_m_ as a function of the peak *I*_Ca_ using the synaptic transfer function:

(2) $$\Delta C_{m}=cI_{\text{Ca}}^{N}$$

where *c* is a scaling coefficient and the power is *N*. The average *N* values reported are from fits to all individual cells tested.

The fusion of vesicles from kinetically distinct vesicle pools was obtained by measuring Δ*C*_m_ in response to depolarizing voltage steps to around − 10 mV, from the holding potential of around −80 mV, of varying duration (2 ms to 1 s). Stimulus durations of up to 100 ms generally allow the isolation of the RRP of vesicles when experiments are performed at body temperature and using 1.3‐mM extracellular Ca^2+^ (Johnson et al., 2010). The size and release kinetics of the isolated RRP were approximated by fitting the data points from each individual cell using a single exponential function. The number of vesicles was estimated using a conversion factor of 37 aF/vesicle (Lenzi, Runyeon, Crum, Ellisman, & Roberts, 1999). Endocytosis is not expected to affect the RRP and SRP measured since in VHCs it is a slow process with an average time constant greater than 8 s (Dulon et al., 2009).

Membrane potentials were corrected for the voltage drop across the series resistance (*R*_s_) (control: 8.4 ± 0.7 MΩ, *n* = 17; *Syt‐4* ^−/−^: 8.0 ± 0.7 MΩ,* n* = 16) and a liquid junction potential of –11 mV using the Cs‐glutamate‐based solution, or –4 mV using the CsCl‐based intracellular solution, measured between electrode and bath solutions. The average size of Type II VHCs as indicated from the whole cell membrane capacitance (*C*_m_) was 4.0 ± 0.2 pF (*n* = 17) in control cells and 4.1 ± 0.1 pF (*n* = 16) in *Syt‐4* ^‐/‐^ cells. All animals were genotyped as previously described (Ferguson et al., 2000). To assess data for statistical significance, we used Student's two‐tailed *t*‐test or, to compare data sets from the two different genotypes, two‐way ANOVA with a multiple comparisons test. Mean values are quoted ± s.e.m. where *p* < .05 indicates statistical significance. The results obtained from *Syt‐4^+/+^* and *Syt‐4^+/−^* VHCs were not significantly different and so were pooled together as the control group for comparisons with *Syt‐4^−/−^* cells.

## 3 RESULTS

### 3.1 Calcium current in control and *Syt‐4^−/−^* mouse utricular Type II VHCs

**Figure 1:**
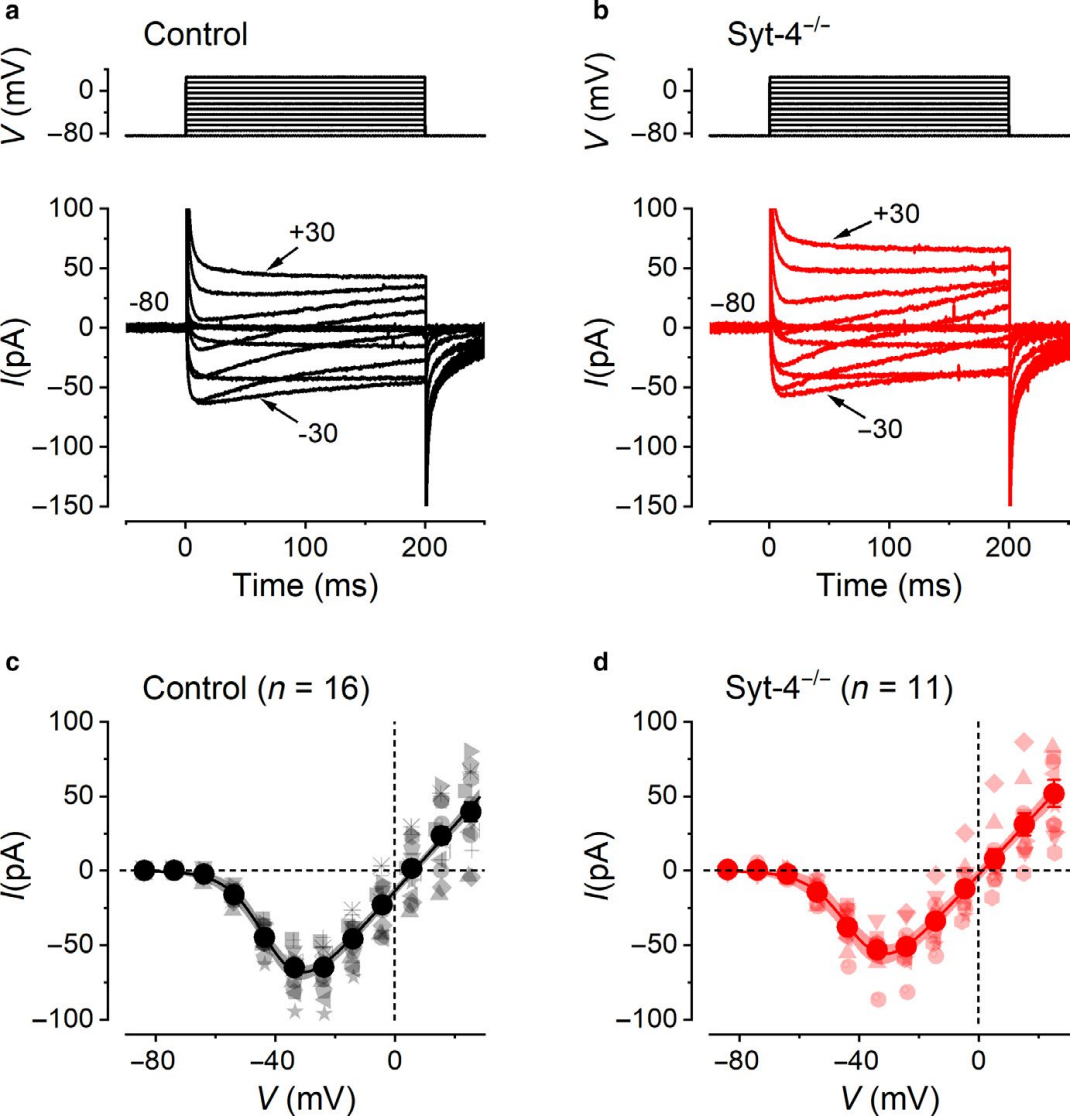
Voltage‐dependent properties of *I*_Ca_ in control and *Syt‐4^−/−^* Type II VHCs. (a) and (b), show average *I*_Ca_ traces (lower panels) recorded from control and *Syt‐4^−/−^* VHCs, respectively. The voltage protocol is shown in the upper panels. *I*_Ca_ was obtained in response to 200 ms voltage steps, in 10 mV increments, from the holding potential of around –80 mV to the membrane voltages shown near to some of the traces (indicated with arrows). Residual capacitive transients have been blanked and leak currents subtracted offline (see Methods). (c and d), show the mean peak *I*‐*V* relation for control (*n* = 16) and *Syt‐4^−/−^* (*n* = 11) VHCs, respectively, obtained by plotting the peak inward current against the membrane potential. Data points from individual cells are shown in light varying symbols and average values are shown in large darker colored filled circles. The solid curves are fits to the individual cell data using Equation 1 (see Methods) and the shaded areas above and below these curves represent the 95% confidence limits of the fit

Although the full complement of voltage‐gated Ca^2+^ channels in VHCs is still largely unknown, the Ca_V_1.3 Ca^2+^ channel subunit is likely to carry the larger component of the total *I*_Ca_ (Bao et al., 2003; Dou et al., 2004; López et al., 1999; Masetto, Zampini, Zucca, & Valli, 2005). Representative *I*_Ca_ traces from control (*Syt‐4^+/+^* and *Syt‐4^+/−^*) and *Syt‐4* knockout (*Syt‐4^−/−^*) Type II VHCs are shown in Figure 1a and 1b, respectively. *I*_Ca_ activated rapidly, reaching a peak within a few milliseconds at most voltages, and then slowly decayed. The mean current‐voltage (*I*‐*V*) relations were obtained by plotting the peak inward current against membrane potential for control and *Syt‐4^−/−^* Type II VHCs (Figure 1c and 1d, respectively). The maximal peak *I*_Ca_ was usually reached at around –30 mV. On average, the maximal peak *I*_Ca_ was found to be similar between control (–65.1 ± 3.3 pA, *n* = 16) and *Syt‐4^−/−^* VHCs (–53.2 ± 4.0 pA, *n* = 11; *p* = .42 from two‐way ANOVA Sidak posttest). To characterize the voltage‐dependent activation of *I*_Ca_, each *I‐V* relation was fitted with Equation 1 (see Methods) (Figure 1c and d). Fits gave similar values between control (*g*_max_, 2.2 ± 0.1 nS; *V*_½_, –40.9 ± 1.1 mV; *S*, 6.4 ± 0.7; *V*_rev_, 6.1 ± 0.7 mV) and *Syt‐4^−/−^* VHCs (*g*_max_, 2.2 ± 0.1 nS; *V*_½_,–40.4 ± 1.8 mV; *S*, 6.7 ± 1.1; *V*_rev_, 1.2 ± 1.0 mV).

In all cases, the *V*_rev_ was not as positive as would be expected from a pure *I*_Ca_ due to the presence of residual Cs^+^ currents through K^+^ channels that were not fully blocked either by perfusion of extracellular K^+^ channel blockers or by their inclusion in the intracellular solution. The residual Cs^+^ currents, however, did not affect the *I*_Ca_ magnitude due to their delayed activation for values up to around the peak of the I‐V curve and only impacted on the values toward more positive potentials. This delayed‐activating Cs^+^ current would also give a false impression of an apparent inactivation of the *I*_Ca_ that is evident, to a similar extent, in the recordings shown in Figure 1a and b. This is why we used the peak *I*_Ca_ values as a measure of *I*_Ca_ size, instead of using the total Ca^2+^ entry that can be approximated using the time integral of the current during the entire voltage step.

### 3.2 Calcium‐dependent exocytosis in control and *Syt‐4^−/−^* Type II VHCs

**Figure 2:**
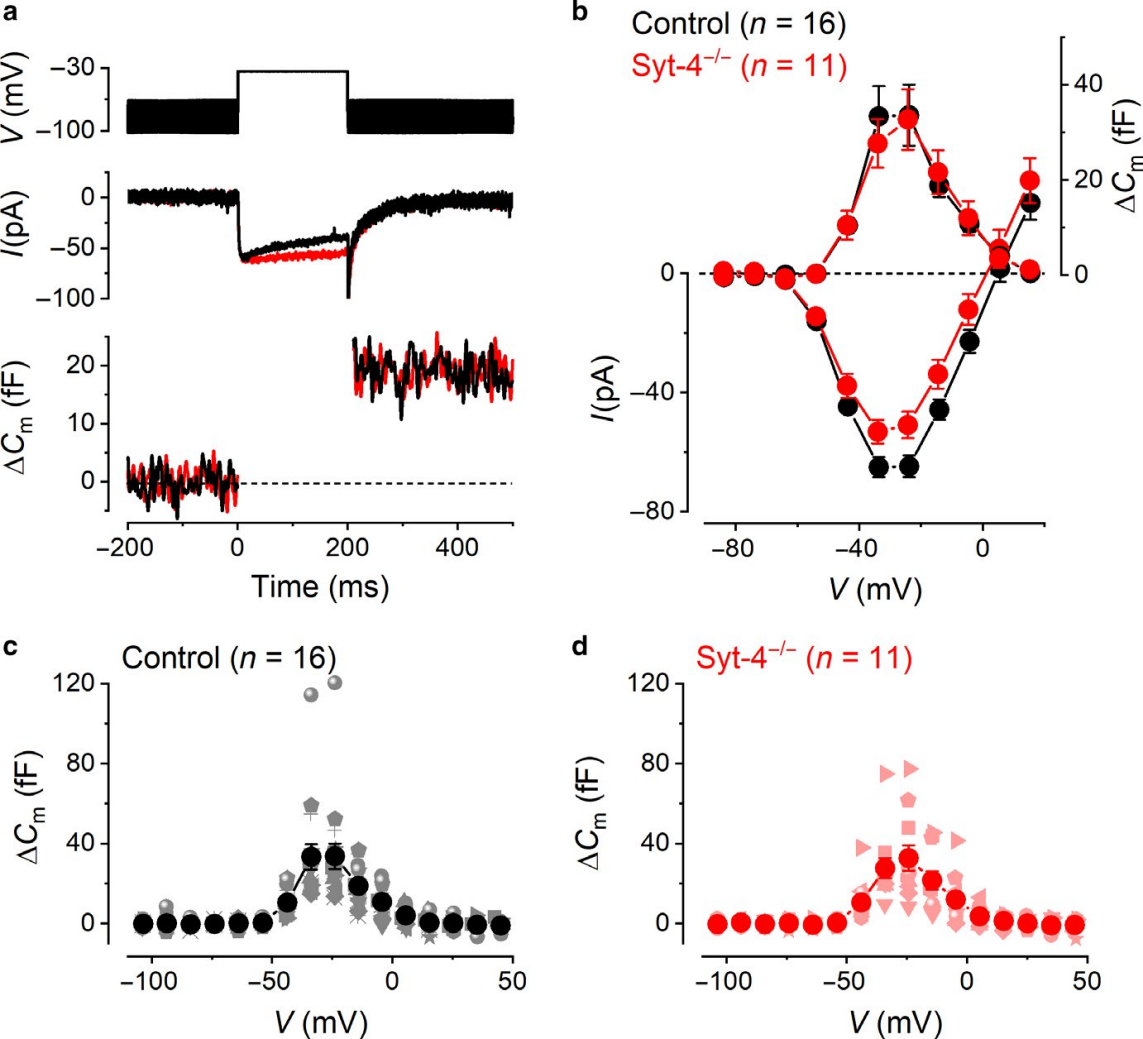
Ca^2+^‐dependent exocytosis in control and *Syt‐4^−/−^* Type II VHCs. (a) representative *I*_Ca_ (middle panel) and Δ*C*_m_ (lower panel) recordings from a control (black trace) and a *Syt‐4^−/−^* (red trace) VHC in response to a 200 ms voltage step to –34 mV from an holding potential of –84 mV. Top panel shows the command protocol applied to the VHCs consisting of a sine wave (thick solid line) interrupted for the duration of the depolarizing voltage step. (b) average *I*‐*V* (lower left‐hand axis; average values are the same as those in Figure 1) and corresponding Δ*C*_m_‐*V* (upper right‐hand axis) curves from control (*n* = 16) and *Syt‐4^−/−^* (*n* = 11) VHCs. (c and d) Δ*C*_m_ data points from individual control and *Syt‐4^−/−^* cells, respectively, are shown by the lighter colored symbols that vary in shape for each cell. Average values are shown as large darker colored filled circles as in (b)

To investigate the possible role of Syt‐4 as a Ca^2+^ sensor for synaptic vesicle fusion at Type II VHC ribbon synapses, exocytosis was monitored by measuring the ∆*C*_m_ triggered by the influx of Ca^2+^ during depolarizing voltage steps of around 200 ms. The fusion of synaptic vesicles to the plasma membrane results in a ∆*C*_m_ that is ultimately considered to be a reflection of the number of vesicles that have fused to the plasma membrane causing the release of neurotransmitter onto afferent nerve terminals (von Gersdorff, Sakaba, Berglund, & Tachibana, 1998; Moser & Beutner, 2000; Neher & Marty, 1982). Typical *I*_Ca_ and corresponding ∆*C*_m_ evoked by a 200 ms voltage step in a control and a *Syt‐4^−/−^* Type II VHC are shown in Figure 2a. On average, depolarization to around –30 mV evoked a maximal ∆*C*_m_ increase of 33.6 ± 6.4 fF (*n* = 16) in control cells, which was similar to the ∆*C*_m_ of 32.7 ± 6.4 fF (*n* = 11) measured in *Syt‐4^−/−^* cells (Figure 2b). The average ∆*C*_m_ values follow a similar bell‐shaped voltage dependence as the corresponding average *I*_Ca_ values (Figure 2b). The average ∆*C*_m_ values obtained at different membrane potentials, in response to 200 ms voltage steps, are plotted along with the individual data points from each cell for control (Figure 2c) and *Syt‐4^−/−^* VHCs (Figure 2d). The Ca^2+^ efficiency of exocytosis, defined as the ratio between ∆*C*_m_ and the *I*_Ca_ (the peak *I*_Ca_ amplitude measured at around –30 mV), was also similar between control and *Syt‐4^−/−^* cells (control: 0.53 ± 0.09 fF/pA, (*n* = 16); *Syt‐4^−/−^*: 0.66 ± 0.13 fF/pA, (*n* = 11)).

### 3.3 Synaptic transfer functions in control and *Syt‐4^−/−^* Type II VHCs

**Figure 3:**
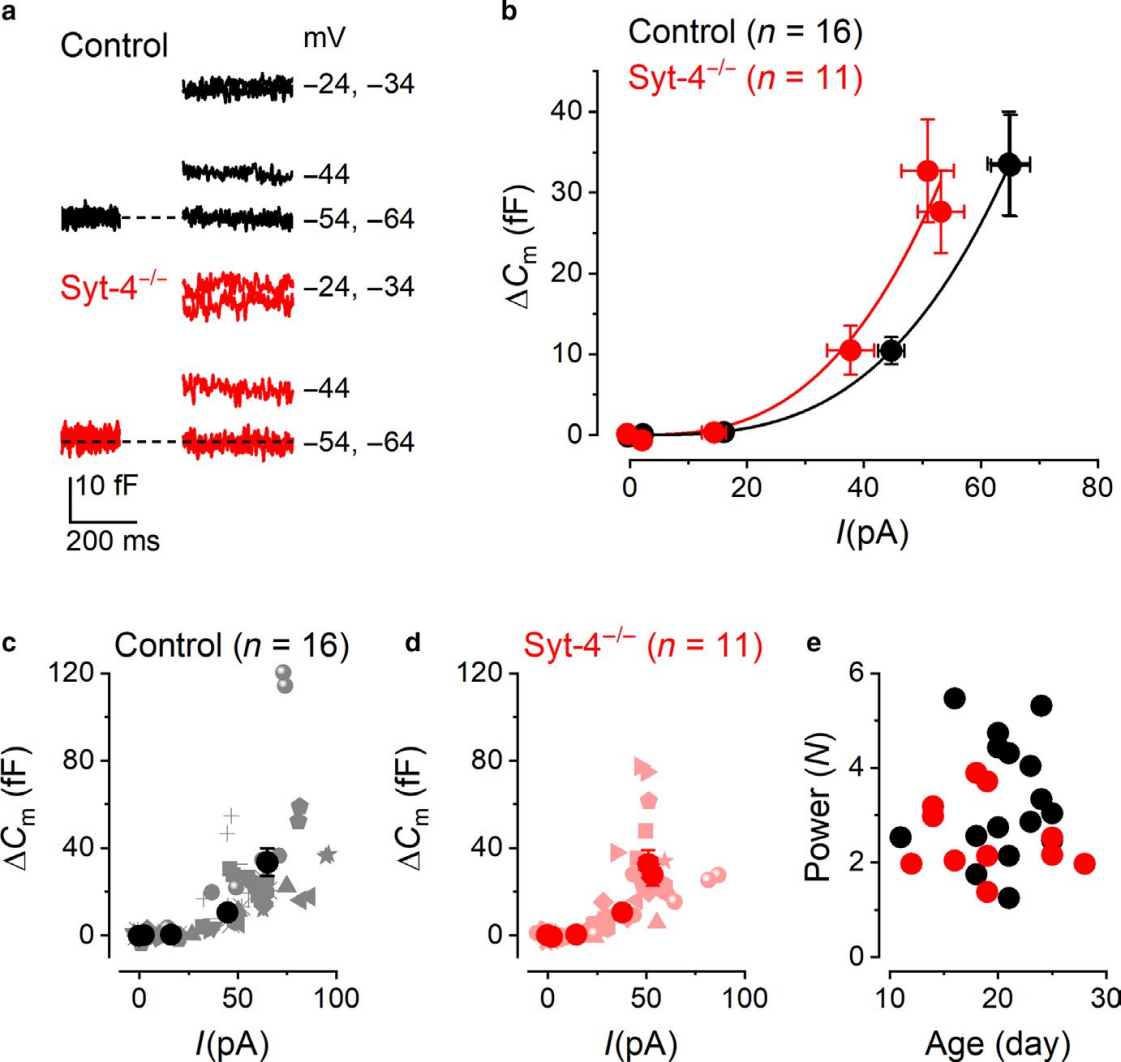
Synaptic transfer functions in control and *Syt‐4^−/−^* VHCs. (a) average Δ*C*_m_ traces from control (black traces) and *Syt‐4^−/−^* (red traces) VHCs recorded in response to 200‐ms depolarization to the different test potentials shown next to the traces. (b) synaptic transfer curves obtained by plotting the average Δ*C*_m_ values against the average corresponding *I*_Ca_ recorded between around –70 mV and –20 mV. Average data points were fitted using a power function (Equation 2). (c and d) Δ*C*_m_ data points plotted against their respective peak *I*_Ca_ value from individual control and *Syt‐4^−/−^* cells, respectively. Data from individual cells are shown by the lighter colored symbols that vary in shape for each cell. Average values are shown as large darker colored filled circles as in (b). (e) Power values from all individual control (black) and *Syt‐4^−/−^* (red) cells plotted against the age of the animal in postnatal day

To assess the role of Syt‐4 in Ca^2+^‐dependent exocytosis of Type II utricular hair cells, the relation between the Ca^2+^ influx and **∆** *C*_m_ in control and *Syt‐4^−/−^* VHCs was estimated using a synaptic transfer function (Augustine et al., 1985). Average **∆** *C*_m_ traces recorded from control and *Syt‐4^−/−^* VHCs in response to Ca^2+^ entry evoked at different test potentials from the holding potential of around –80 mV are shown in Figure 3a. Synaptic transfer functions for control and *Syt‐4^−/−^* VHCs were obtained by plotting the average ∆*C*_m_ values against the peak *I*_Ca_ over a physiological range of potentials between around –70 mV and –20 mV (Figure 3b). The peak *I*_Ca_ was used instead of charge integral (Johnson et al., 2010) in order to minimize any possible error caused by the unblocked outward current (Figure 1). The individual ∆*C*_m_ and *I*_Ca_ data points from each cell are shown, along with the average values, for control (Figure 3c) and *Syt‐4^−/−^* VHCs (Figure 3d).

The average power obtained from fitting the transfer function of individual VHCs using Equation 2 (see Methods) was found to be similar between the two genotypes (control: *N* = 3.3 ± 0.3, *n* = 16; *Syt‐4^−/−^*: *N* = 2.5 ± 0.2, *n* = 11; *p* = .09 two‐tailed *t*‐test). These results show that there is a supra‐linear relation between Ca^2+^ influx and neurotransmitter exocytosis in mature VHCs, suggesting a cooperative process requiring multiple Ca^2+^ binding steps (Augustine et al., 1985; Dodge & Rahamimoff, 1967; Dudel, 1981; Zucker, 1985, 1993). The power obtained from individual Type II VHCs from both control and *Syt‐4^−/−^* mice showed no developmental trend over the age range of animals investigated (Figure 3e).

### 3.4 Kinetics of vesicle pool release in control and *Syt‐4^−/−^* VHCs

**Figure 4:**
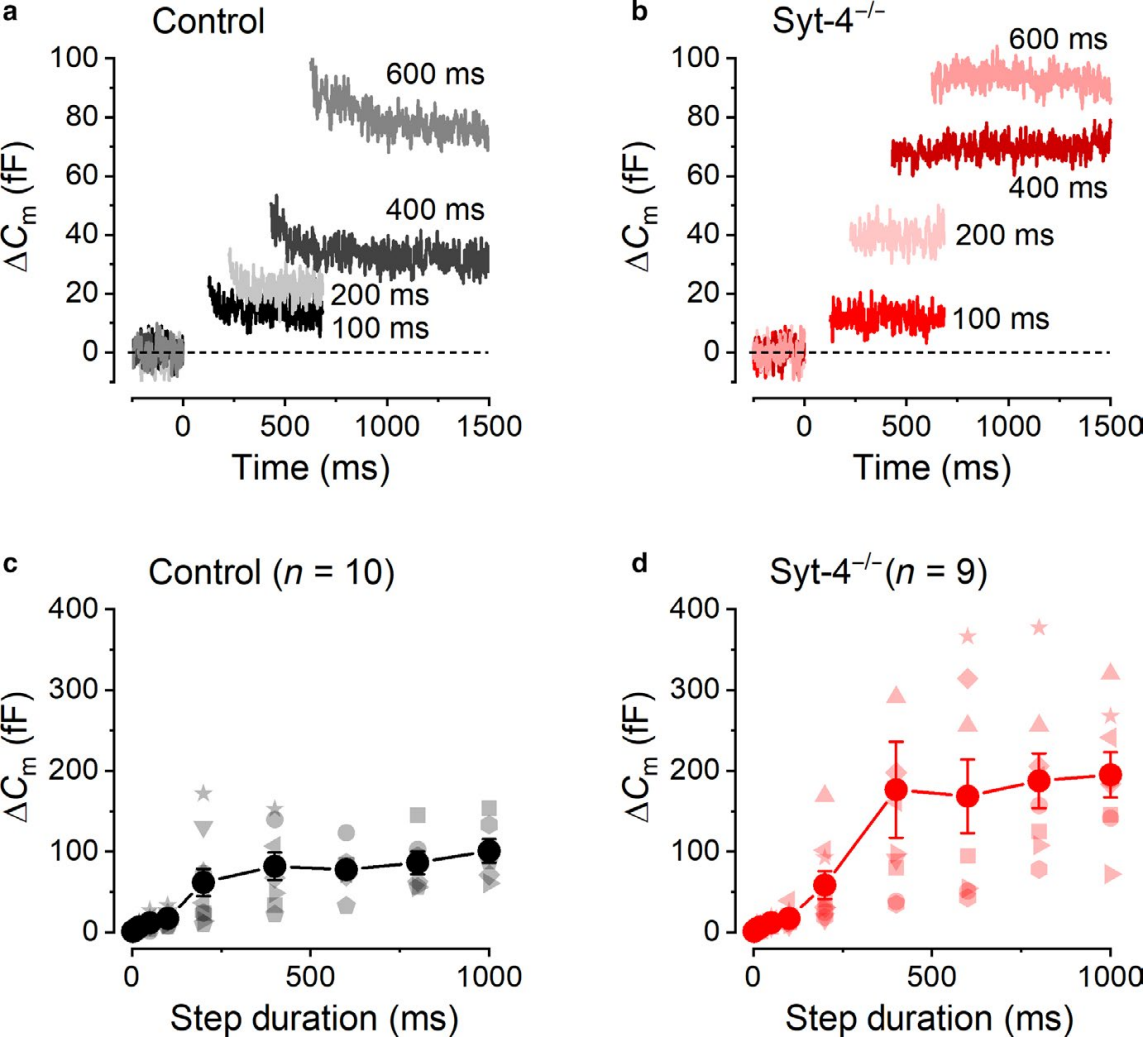
Neurotransmitter release from distinct vesicle pools in control and *Syt‐4^−/−^* VHCs. (a and b), representative Δ*C*_m_ recordings from a control (a) and a *Syt‐4^−/−^* (b) VHC in response to voltage steps of different duration (indicated next to the traces) to around –10 mV from a holding potential of –80 mV. (c and d) average ∆*C*_m_ values obtained from control (*n* = 10) and *Syt‐4^−/−^* (*n* = 9) VHCs in response to depolarizing voltage steps to − 10 mV of increasing duration (from 2 ms to 1,000 ms). Data from individual cells are shown by the lighter colored symbols that vary in shape for each cell and average values are shown as large darker colored filled circles

Possible changes in the dynamics of vesicle pool recruitment due to a lack of Syt‐4 in Type II VHCs were investigated by measuring ∆*C*_m_ in response to depolarizing voltage steps to around –10 mV that increased in duration from 2 ms to 1 s. In 1.3‐mM extracellular Ca^2+^, the shorter stimuli up to around 100 ms evoke ∆*C*_m_ that are usually ascribed to the RRP of vesicles docked at the active zones of the presynaptic membrane (Moser & Beutner, 2000). Longer stimuli trigger the release of an additional, larger and slower ∆*C*_m_ component, associated with a SRP of vesicles that are likely to be tethered further up the ribbon away from the active zones (von Gersdorff & Matthews, 1999; von Gersdorff, Vardi, Matthews, & Sterling, 1996; Voets, Neher, & Moser, 1999). The ∆*C*_m_ responses from a control and a *Syt‐4^−/−^* Type II VHC to depolarizing voltage steps of different durations are shown in Figure 4a and 4b, respectively. The average ∆*C*_m_ as a function of the depolarizing step duration for control (*n* = 10; Figure 4c) and *Syt‐4^−/−^* (*n* = 9; Figure 4d) VHCs indicates that there is an initial foot region corresponding to the release of the RRP during the first 100 ms of stimulation, which is followed by a larger release of the secondary pool of vesicles for voltage steps between 200 ms and 1,000 ms.

**Figure 5:**
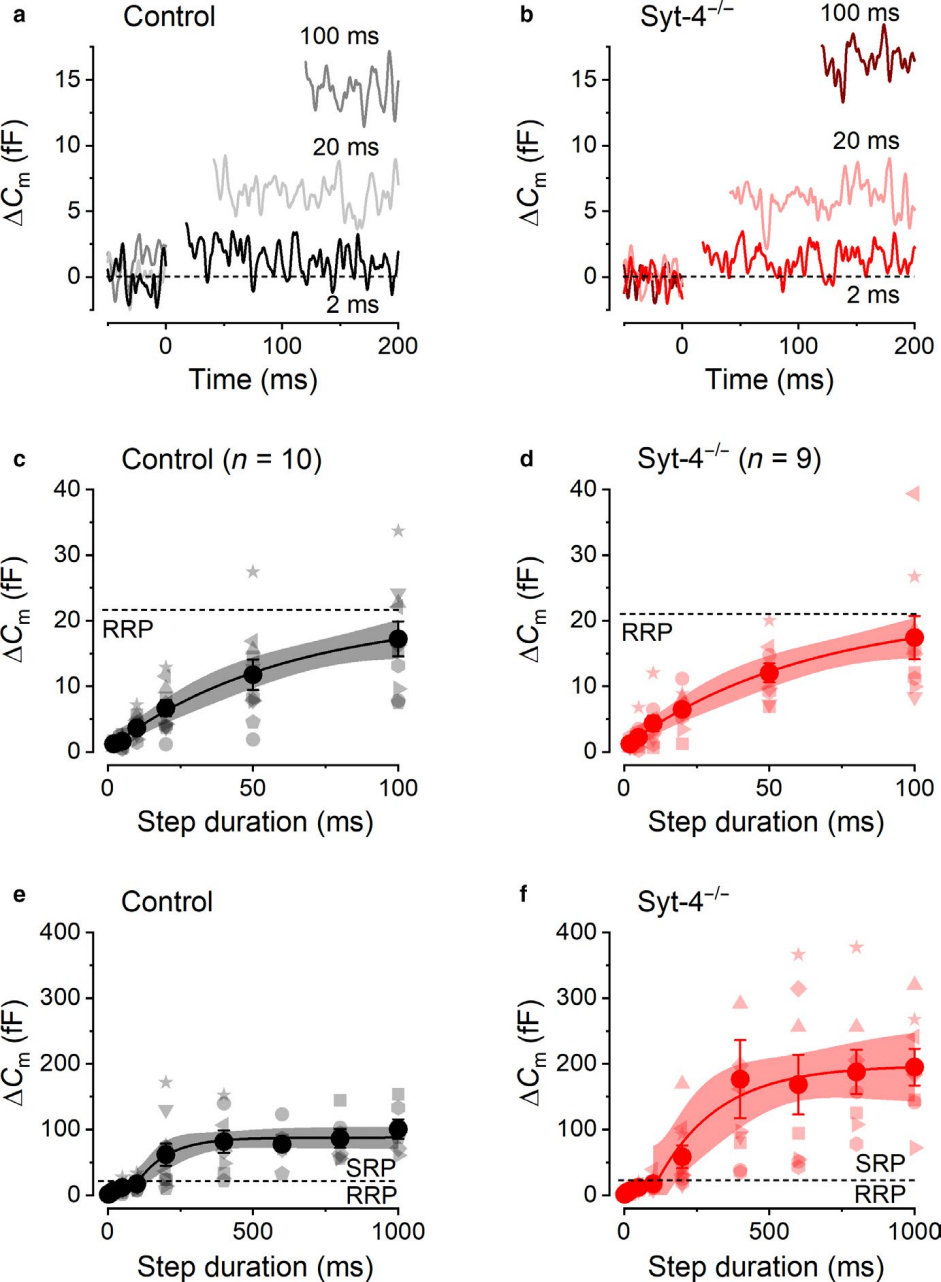
The kinetics of RRP and SRP exocytosis in control and *Syt‐4^−/−^* VHCs. (a) and (b), average Δ*C*_m_ recordings from control (a) and *Syt‐4^−/−^* (b) VHCs in response to –10 mV voltage steps of up to 100 ms in duration (indicated next to the traces) that trigger the release of the RRP of synaptic vesicles. (c) and (d), average ∆*C*_m_ values as in Figure 4c and 4d showing the responses to depolarizing voltage steps to −10 mV from 2 ms up to 100 ms. Data from individual cells are shown by the lighter colored symbols and averages as large darker colored filled circles. The distributions of individual ∆*C*_m_ responses recorded from individual control and *Syt‐4^−/−^* VHCs were fitted with a mono‐exponential function (solid curves) and the shaded areas above and below these curves represent the 95% confidence limits of the fits. The dashed horizontal lines represent the maximal ∆*C*_m_ values obtained from the exponential fits and therefore delineate the size of the RRP. (e) and (f), show the same data as in Figure 4c and d and ∆*C*_m_ data points in the SRP from individual cells have been fit with mono‐exponential functions and are shown as solid curves. Values from 200 ms to 1,000 ms were used for the fitting. The shaded areas above and below these curves represent the 95% confidence limits of the fits

The RRP of vesicles was recruited by depolarizing step lengths up to around 100 ms (Figure 5a‐d). This initial kinetic component of vesicle release could be well approximated using a single exponential function. Average ∆*C*_m_ traces in response to voltage steps up to 100 ms for control and *Syt‐4^−/−^* VHCs are shown in Figure 5a and 5b, respectively. Fitting the average RRP data (Figure 5c and 5d) revealed a similar maximal ∆*C*_m_, for this initial component, of 21.5 ± 6.2 fF (*n* = 10) in control and 21.1 ± 5.6 fF (*n* = 9) in *Syt‐4^−/−^* VHCs (*p* = .963 two‐tailed *t*‐test). Using a conversion factor of 37 aF per vesicle (Lenzi et al., 1999), the maximal ∆*C*_m_ values obtained from the fits gave a total number of about 580 vesicles in the RRP for both control and *Syt‐4^−/−^* VHCs. The time constant of RRP release was also similar in control (62.2 ± 37.9 ms, *n* = 10) and *Syt‐4^−/−^* VHCs (60.1 ± 34.0 ms, *n* = 9; *p* = .967 two‐tailed *t*‐test). These values gave similar initial release rates for the RRP (control: 346 fF/s or ~ 9,350 vesicles/s; *Syt‐4^−/−^*: 351 fF/s or ~ 9,490 vesicles/s). Considering a mean of 7 to 9 ribbons per VHC (Dulon et al., 2009), the number of RRP synaptic vesicles per ribbon (SV/ribbon) was estimated to be 64–83 SV/ribbon for both control and *Syt‐4^−/−^* VHCs. These results were consistent with those reported previously in wild‐type VHCs (Dulon et al., 2009) and mature mouse cochlear IHCs (Nouvian, Beutner, Parsons, & Moser, 2006). Taken together, these results suggest that the vesicles released from the RRP in Type II VHCs of mice lacking Syt‐4 have similar kinetic properties to those in control VHCs.

While there were no significant differences among ∆*C*_m_ within the RRP, the values were significantly larger in *Syt‐4^−/−^* cells compared to controls for values within the SRP between 400 ms and 1,000 ms (two‐way ANOVA Sidak posttest; 400 ms *p* < .01; 600–1000 ms *p* < .05). Fitting the SRP data, separately from the RRP, with single exponential functions (Figure 5e and 5f) revealed a different maximal average ∆*C*_m_ for this secondary component of 70.7 ± 8.3 fF (*n* = 10) in control and 180.2 ± 31.8 fF (*n* = 9) in *Syt‐4^−/−^* VHCs (*p* < .005 two‐tailed *t*‐test). The maximal ∆*C*_m_ values obtained from the fits gave a total number of about 1910 and 4,870 vesicles in the SRP for control and *Syt‐4^−/−^* VHCs, respectively, giving an estimated apparent number of SRP synaptic vesicles per ribbon as 210–270 for control and 540–695 for *Syt‐4^−/−^* VHCs. The maximal size of the SRP in control Type II VHCs of around 100 fF is similar to that reported previously in control Type II VHCs (Dulon et al., 2009). Although the size of the SRP was apparently smaller in control Type II VHCs than in *Syt‐4^−/−^*, the time constant of release was faster (106.4 ± 57.4 ms, *n* = 10) than in *Syt‐4^−/−^* VHCs (213.9 ± 134.9 ms, *n* = 9; *p* = .457 two‐tailed *t*‐test) but was not significantly different. These values gave more comparable initial release rates for the SRP between the two genotypes (control: 663 fF/s or ~ 17,910 vesicles/s; *Syt‐4^−/−^*: 842 fF/s or ~ 22,770 vesicles/s).

## 4 DISCUSSION

We found that the Ca^2+^ dependence of neurotransmitter release is high‐order in mature Type II mouse VHCs using physiological recording conditions (1.3‐mM extracellular Ca^2+^ and body temperature). Syt‐4 was not involved in determining the Ca^2+^ dependence of synaptic vesicle exocytosis nor in the kinetic properties of the release of the RRP of vesicles in mature Type II VHCs, which is in agreement with the presence of Syt‐4 being correlated only with the linear exocytotic Ca^2+^ dependence in mature mouse cochlear IHCs. However, we did notice that the SRP of vesicles was larger in mice lacking Syt‐4, which could suggest a role for Syt‐4 in regulating vesicle release during prolonged stimulation in these cells. By comparison with auditory cells, it appears that the presynaptic features of VHCs are a specialization for encoding very low‐frequency signals.

### 4.1 Syt‐4 is not involved in RRP exocytosis in Type II VHCs

The identity of the Ca^2+^ sensor at VHC synapses has been less thoroughly investigated than in cochlear IHCs, where otoferlin has been proposed as the main Ca^2+^ sensor for exocytosis (Roux et al., 2006), as well as being implicated in many other aspects of IHC synaptic transmission (e.g., vesicle pool replenishment: Johnson et al., 2010; Pangrsic et al., 2010). Otoferlin has been shown to be expressed in both types of VHC; however, its importance in vesicle release was less obvious than in cochlear hair cells (Dulon et al., 2009). The spontaneous release of neurotransmitter in VHCs, which presumably uses the same synaptic machinery at least in Type II cells, was shown to still be present in *otoferlin* knockout mice (Dulon et al., 2009). Instead, otoferlin was implicated in determining the linear Ca^2+^ dependence of vesicle release in VHCs (Dulon et al., 2009). While otoferlin is an essential component of the Ca^2+^‐sensing machinery at mammalian IHC ribbon synapses (Roux et al., 2006), the linearization of the Ca^2+^ dependence at around the onset hearing has been shown to be dependent on the presence of the Ca^2+^ sensor Syt‐4 in high‐frequency cells (>2–3 kHz; Johnson et al., 2010). Syt‐4 was not observed in cells that show a high‐order exocytotic Ca^2+^ dependence, such as immature IHCs or very low‐frequency (<2–3 kHz) gerbil IHCs (Johnson et al., 2010). Note that mice, different from gerbils, do not poses verylow‐frequency IHCs and all mature cochlear IHCs show a linear coupling between *I*_Ca_ and exocytosis. Using *Syt‐4^−/−^* mice, we found that Syt‐4 plays no functional role in determining the Ca^2+^ dependence or kinetics of vesicle release from the RRP in mature Type II VHCs, which process stimuli in the comparatively very low‐frequency range of 0.1–50 Hz (Grossman, Leigh, Abel, Lanska, & Thurston, 1988). We found that the Ca^2+^ dependence of exocytosis was high‐order in both control and *Syt‐4^−/−^* cells, with a power value of around 3. This further supports the view that Syt‐4 determines the linear coupling between *I*_Ca_ and exocytosis only in higher frequency hair cells, where a linear coupling would ensure that exocytosis is proportionate to the gradual variation of the receptor potential magnitude with stimulus intensity.

The molecules determining the Ca^2+^ dependence of vesicle release at VHC ribbon synapses are less well understood than they are in cochlear hair cells. The absence of a functional role for Syt‐4 in determining the Ca^2+^ dependence of RRP release in Type II VHCs suggests that it is determined by otoferlin alone or possibly in combination with other Ca^2+^‐sensing molecules such as other isoforms of synaptotagmin (Sudhof, 2002). The high‐order Ca^2+^ dependence suggests that Syt‐1 or Syt‐2 could be involved, especially considering that these isoforms are expressed in immature cochlear IHCs that show a similar high‐order Ca^2+^ dependence (Beurg et al., 2010; Johnson et al., 2010; Reisinger et al., 2011). This also seems plausible considering that otoferlin is unlikely to account for all VHC vesicle fusion because of the continued presence of spontaneous release from VHCs in *otoferlin* knockout mice together with the lack of clear vestibular phenotype in these animals (Dulon et al., 2009; Roux et al., 2006).

The high‐order Ca^2+^ dependence of synaptic vesicle exocytosis observed here in mature mouse Type II VHCs (P11‐P28) differs from that previously reported (Dulon et al., 2009), where neurotransmitter exocytosis was found to be linearly coupled to Ca^2+^ influx in immature VHCs (P4‐P9). This difference could arise from the immature nature of the afferent terminals and synaptic contacts on VHCs at P4‐P9 (Rüsch, Lysakowski, & Eatock, 1998). It is also possible that the larger single‐channel Ca^2+^ inflow resulting from the higher extracellular Ca^2+^ concentration used by Dulon et al. (2009) saturates the vesicle Ca^2+^ sensor when a nearby Ca^2+^ channel opens. This might easily occur if exocytosis of a synaptic vesicle is mainly controlled by one or few Ca^2+^ channels located in nanometer proximity to the release site of a vesicle (Neher, 1998). A nanodomain coupling between Ca^2+^ entry and synaptic vesicle release was reported for Type II VHCs (Dulon et al., 2009) and for verylow‐frequency adult gerbil auditory IHCs (Johnson, Olt, Cho, von Gersdorff, & Marcotti, 2017). Another similarity between Type II VHCs and verylow‐frequency gerbil IHCs is that both have spherical synaptic ribbons (Favre & Sans, 1979; Johnson et al., 2008) as opposed to the more elongate ribbons of mature higher frequency hair cells (Johnson et al., 2008; Khimich et al., 2005; Sobkowicz, Rose, Scott, & Slapnick, 1982). The wider base of the spherical ribbons could ensure a closer coupling between Ca^2+^ channels and docked vesicles and/or act as a diffusion barrier for Ca^2+^ such that the concentration rapidly increases around the release sites (Graydon, Cho, Li, Kachar, & von Gersdorff, 2011).

### 4.2 Functional relation between Type II VHC synaptic transmission and afferent activity

The nonlinear coupling between *I*_Ca_ and exocytosis in mature Type II VHCs found here, combined with a tight nanodomain coupling of Ca^2+^ channels and vesicle release sites (Dulon et al., 2009), would ensure synaptic transmission with the rapid latency required for driving vestibular‐ocular and balance reflexes. It has been suggested that a similar high‐order Ca^2+^ dependence in apical (verylow‐frequency) gerbil IHCs could accentuate the onset of the receptor potential to accurately localize verylow‐frequency sounds (Johnson, 2015; Johnson et al., 2017). However, extrapolation to the vestibular system is complicated by the evidence that each vestibular afferent can make contact with several hair cells, whereas each auditory afferent only makes one synaptic contact with a single IHC. It is possible that the vestibular innervation pattern is designed to favor sensitivity over fine tuning. Indeed, vestibular organs are exquisitely sensitive to small head movements (Wilson & Melville‐Jones, 1979). Sensitivity might also take advantage from a supra‐linear coupling with multiple VHCs, where cells with different thresholds become progressively recruited by stimuli of increasing intensity. In this case, each VHC would provide a substantial contribution to the total afferent signal when specifically activated by displacement of the otolithic membrane (or *cupula*) and its threshold level is reached. Since the relation between stimulus intensity and utricular afferent fiber discharge is believed to be linear within the range of accelerations typically experienced in normal life (Fernandez & Goldberg, 1976; Goldberg, Lysakowski, & Fernández, 1990), the summation of nonlinear components of different threshold could give the appearance of an overall linear relation. On the other hand, it could also be possible that each VHC contributes to the whole range of stimulus magnitudes, that is the total afferent response results from the product of *n* cells contacted. In the latter case, the supra‐linear increase of vesicle fusion with *I*_Ca_ might compensate for sublinear component/s in the signal transduction cascade, which again results in an overall linear relation. For example, the motion of the otolithic membrane might tend to saturate over large stimuli, as shown for *cupula* deflection in semicircular canals (Wilson & Melville‐Jones, 1979). Interestingly, we found that the SRP of vesicles was larger in mice lacking Syt‐4, which could suggest a role for Syt‐4 in lowering vesicle release during prolonged stimulation in these cells, thus limiting excessive neurotransmitter exocytosis. It is generally considered that Syt‐4 is an inhibitory isoform due to its inability to bind Ca^2+^ in the C_2_A domain (Ullrich et al., 1994), and it has been shown to negatively regulate vesicle exocytosis in PC12 cells and neurons (Dean et al., 2009; Machado, Liu, Vician, & Herschman, 2004). The normal Ca^2+^ regulation and RRP kinetics but increased size of the SRP observed in *Syt‐4^−/−^* Type II VHCs is consistent with previous findings where Syt‐4 promoted exocytosis for low levels of Ca^2+^ influx but inhibited at high Ca^2+^ levels in pituitary nerve terminals (Zhang, Bhalla, Dean, Chapman, & Jackson, 2009).

A difference in size of the SRP could imply that synaptic ribbons, that act as a store of vesicles and create the SRP (Matthews & Fuchs, 2010), would either be more numerous or larger in *Syt‐4^−/−^* VHCs. This, however, seems unlikely since Syt‐4 has not been shown to have a role in ribbon structural architecture and also the fact that the RRP size and kinetics are the same in these cells implies that the number and size of ribbons are the same. Alternatively, the different SRP values obtained could be caused by variation between the sampled cells since the size of the SRP was more variable between cells than that of the RRP.

## CONFLICT OF INTEREST

The authors declare no conflict of interest.

## AUTHOR CONTRIBUTIONS

PS and SLJ performed the experiments, analyzed the data, and wrote the manuscript. All authors came up with the idea for the study and designed the experiments. SM and WM helped to write the manuscript.

## Data Availability

The raw data supporting the conclusions of this manuscript will be made available by the authors, without undue reservation, to any qualified researcher.
